# Editorial: Emerging Topics in Dietary Assessment

**DOI:** 10.3389/fnut.2019.00176

**Published:** 2019-11-19

**Authors:** Aida Turrini, Laura D'Addezio, Emily Dhurandhar, Marika Ferrari, Cinzia Le Donne, Lorenza Mistura, Raffaela Piccinelli, Maria Luisa Scalvedi, Stefania Sette

**Affiliations:** ^1^Council for Agricutural Research and Economics, Research Centre for Food and Nutrition, Rome, Italy; ^2^Department of Kinesiology & Sport Management, Texas Tech University, Lubbock, TX, United States

**Keywords:** dietary assessment methodology, food frequency questionnaire (FFQ), sodium and potassium intake, food atlas, portion size, automated food matching, self-reporting plausibility, diet-disease relationship

Papers in the present editorial project highlight various aspects of new issues and topics, new methodologies, and new technologies, which have become increasingly important in recent years.

New technologies allow researchers to reduce the burden of participants and surveyors. To obtain reliable results is questionable if it is necessary to address scientific general topics such as proper survey design. Managing non-random errors is crucial for accuracy because it limits uncertainty and makes it possible to identify new issues to analyse.

Gazan et al. addresses methodological issues by designing a suitable tool for investigating dietary patterns on a large scale and develops a validated food frequency questionnaire. The following article (Athanasatou et al.) is focused on validating indicators for dietary adequacy evaluation.

Data management and processing topics such as (a) data quality from underpinning databases (nomenclatures and food information—food description and coding, portion sizes, “working” food composition, quantification tools, e.g., photographic food book, recipes) must be carefully maintained; and (b) procedures for data cleaning (ensuring accuracy), completing (e.g., calculating indexes), food matching and, processing at statistical level (i.e., defining the data matrix and defining the most suitable statistical models) need to be validated and software systems need to be implemented.

The article related to quantification of food intake describes an experience related to building (Nikolić et al.) which represents the main quantification tool as indicated in the EU-Menu methodology (1). Software used to deal with food matching (Lamarine et al.) have been addressed in the paper here published thus contributing to the development of an automated food matching tool as proposed in the EURODISH^1^ project (2).

Investigating new topics related to the increasing adoption of the self-reporting approach is crucial to ensure data reliability. It is discussed in the paper (Banna et al.).

Finally, use of data is another feature which is carefully addressed and analyzed in Archer et al.. The article encourages researchers to continue to develop suitable protocols for new perspectives (3) but also to explain the correct use of epidemiological studies. In fact, the paper clearly highlights that epidemiological data are misused. The set of papers here presented provides starting points for a general reflection on purposes, features, and use of dietary assessment within new perspectives, particularly in building research infrastructures (4).

The main objective in carrying out surveys is to get a large set of information which allows scientists to analyse indicators in a comparative way. In fact, epidemiological studies are aimed at analyzing statistics at the population level for public health nutrition purposes [see e.g., (5) and all the Global Burden of Disease publications^2^]. These statistics can then be used in planning clinical studies that provide scientific values relative to links between diet and disease. Hence, it is recommended that data are collected exactly in the same way to limit influence of accidental factors and personal views (1). Standardization and training are two pillars to achieve this, so statistical analysis of relationships can be performed without distortion. We don't use the word “*bias*” voluntarily as distortion deals with accuracy and uncertainty while bias is a statistical-mathematical concept. The results from epidemiological studies are not absolute. Dietary data from different groups characterized by key factors (age/gender, socio-economical, geographical, physiological conditions, etc.) can be compared to verify statistical associations. Prospective studies can verify associations due to the past (diet history) or future (longitudinal studies) or present (case-control studies) but the principle is the same, non-standardized methods can lead to inaccurate information basis. In this view, an appropriate presentation is crucial to allow for evaluating papers concerning nutritional epidemiological studies (6), and including results in systematic literature reviews and meta-analyses (7). In a research infrastructure perspective the two approaches can be used in a synergic way to appropriately face public health issues.

Some other issues must be tackled in planning dietary assessment studies. For example, food grouping is crucial in order to ensure statistical significance and to make dietary data understandable (8). The FoodEx2, an advanced food coding system, has been developed by the European Food Safety Authority (9) merging approaches to coding systems from European projects on food description (LanguaL^3^) and food aggregation Eurocode2^4^.

Further application of statistical classification techniques could be used to validate aggregation criteria and analyzing aspects in-depth related to the concept of human nutritional sustainability which has recently been introduced (10). Mobile apps increase the need to underpin database management on the input side, while representativeness of population in samples is challenging too. Systems developed in European projects concerning mHealth and eHealth are very important for professionals working in these fields to build various tools such as the QUISPER^5^ platform for dietary advice for personalized nutrition; the Parkinson disease management tool^6^, and PRECIOUS^7^ infrastructure for e-health care. Moreover, the project RICHFIELDS^8^ has been designed to build an infrastructure for managing food and health consumer behavior and lifestyle.

Research infrastructures are expected to make feasible to put in practice FAIR principles, i.e., findability, accessibility, interoperability, and reusability features in open data environment (4, 11).

We would like to encourage investigators to build a consolidated information system while continuing to collect contributions in a hyper-journal tailored for this purpose.

## Author Contributions

AT drafted the manuscript. All co-authors contributed to the manuscript.

### Conflict of Interest

The authors declare that the research was conducted in the absence of any commercial or financial relationships that could be construed as a potential conflict of interest.

## Footnotes

^1^http://www.eurodish.eu/

^2^http://www.healthdata.org/gbd.

^3^https://www.langual.org/default.asp

^4^http://www.danfood.info/eurocode/

^5^http://www.qualify-fp7.eu/

^6^https://ec.europa.eu/programmes/horizon2020/en/news/pdmanager-mhealth-platform-parkinsons-disease

^7^http://www.thepreciousproject.eu/

^8^https://www.richfields.eu/
